# Exploring the contributions of sex and traditionally genderized interpersonal-expressive traits to variability in post-trauma pain ratings

**DOI:** 10.1371/journal.pone.0278399

**Published:** 2022-12-07

**Authors:** Maryam Ghodrati, David M. Walton, Joy C. MacDermid

**Affiliations:** Health and Rehabilitation Sciences Program, University of Western Ontario, London, Canada; University of Catania Libraries and Documentation Centre: Universita degli Studi di Catania, ITALY

## Abstract

**Objectives:**

Multiple intra- and inter-individual variabilities sculpt the experience of pain. However, integration of sex and gender has been under-explored in explanatory models of pain. This study aimed to examine the role of sex and traditionally genderized interpersonal-expressive traits, and their interactions in explaining the variability of pain ratings.

**Methods:**

Data from 113 participants following acute non-catastrophic musculoskeletal (MSK) injuries were included. Participants completed the Brief Pain Inventory (BPI) and the Gender, Pain and Expectations Scale (GPES). An independent T-test was used to compare differences in BPI subscales between the sexes. Pearson correlations explored the associations between BPI and GPES subscale scores for the overall sample and also for the sample when disaggregated by sex. Multiple linear regression was used to investigate the interaction of sex and gender traits in explaining the BPI scores.

**Results:**

No differences were found between the sexes in mean BPI Severity and Interference. Across sexes, Relationship-oriented was positively associated with greater BPI Severity (r = 0.20) and Emotive was positively associated with BPI Interference (r = 0.24). In sex-disaggregated analyses, these associations were significant in females only. Goal-oriented was associated with neither BPI Severity nor Interference. In multivariate regression, only Emotive was a significant predictor of BPI Interference.

**Discussion:**

The findings suggest that variances in pain-related interference are partially explained by scores on a scale measuring self-perceptions of Emotive qualities. Sex was not predictive of either pain outcome in both bivariate and multivariate analyses. Researchers and clinicians are encouraged to consider both sex- and gender-based variables when interpreting patient pain reports.

## Introduction

Sex-based differences have been studied in pain for several decades. For example, prior epidemiological research suggests that women are over-represented compared to men across most musculoskeletal (MSK) pain disorders [1–4]. Similarly, a large volume of laboratory and clinical research reports that females tend to report or exhibit higher pain sensitivity, intensity, and lower pain tolerance than males when exposed to similar pain stimuli [5–8]. Despite the evidence of sex-based differences in pain reporting or experience, there are enough inconsistent findings (e.g. [9, 10]) to suggest that ascribing those differences to a simple dichotomy of biological sex is missing a larger biopsychosocial picture. Various factors from multiple domains of study likely have an interdependent influence on pain [11]. For example, from a biological perspective, empirical evidence exists to suggest the differences between males and females may be due to differences in how nociceptive signals are generated or transmitted. These could be functions of genotypes [12–14], or the sensitivity of nociceptive/pain pathways due to relative sex hormone concentration [7, 15–17]. However, even here, the differences have not been fully or consistently explained based on biology alone.

In human research, pain can only be known as far as individuals are willing and able to express it, thus it is filtered through cognitive and interpersonal states, experiences and values. Less explored are the potential gender-based psychological and social influences on pain experience or reporting. Per the recent revision of the definition of pain and accompanying notes, people learn the concept of pain through life experiences [18], and those experiences appear to interact with personality factors [19]. Accordingly, it has been suggested that person-level characteristics such as introversion or stoicism could act as vulnerability or resilience factors for pain [19, 20]. For example, emotional vulnerability as measured by the Personal Attribute Questionnaire (PAQ) Scale [21], explained sex differences in pain better than catastrophizing, and higher pain reporting / lower pain tolerance in women could be partly explained by differences in emotional vulnerability [22].

Pain reports and behaviours are best conceptualized as communicative tools intended to translate personal experiences into interpersonal messages [23]. As with any behaviour, pain ratings might be shaped by social pressures regarding what is normal and acceptable for a given context or gender. While sex was assigned to explaining biological differences, the CIHR Institute of Gender and Health describes gender as “the socially constructed roles, behaviours, expressions and identities of girls, women, boys, men, and gender diverse people. It influences how people perceive themselves and each other, how they act and interact, and the distribution of power and resources in society. Gender is usually conceptualized as a binary (girl/woman and boy/man) yet there is considerable diversity in how individuals and groups understand, experience, and express it” [24]. Accordingly, most current conceptualizations of gender describe the concept as a continuum on which a person could fall somewhere between exclusively “feminine” or exclusively “masculine” characteristics as prescribed by society [25, 26] though the operationalizations of those terms and the traits that comprise appear to have been poorly defined, reported, or understood in pain research.

Gender roles for men and women have had different definitions in different communities and cultures and have even changed over time [27–29], for example, some societies expect men and women to have different reactions to pain [30, 31]. While the traditional concepts of gender-based norms are being rapidly challenged and transformed in North America, there remain certain interpersonal qualities that are more common to one sex. For example, despite an increase in women in the workforce over the past 50 years, goal-directed leadership qualities are still more commonly ascribed to males [32–36]. Similarly, despite the increasing involvement of males in caregiver roles, interpersonal nurturing and emotive qualities remain more commonly ascribed to females [32, 37].

Whether these traditionally genderized qualities can also explain some of the interindividual variances in pain and disability ratings is an area demanding further study. However, one of the major challenges in quantitative gender-based investigations of pain is the lack of clear consensus on gender constructs and appropriate tools to measure them. The Gender, Pain, and Expectations Scale (GPES) was designed to capture self-rated traditionally genderized traits and to assess gender-based perceptions of personal pain behaviours (pain sensitivity, endurance, reporting). A structural analysis of the genderized traits section of this scale led to the identification of three main subfactors: Relationship-oriented, Goal-oriented, and Emotive which represent areas where society has traditionally placed gendered expectations on women and men [38].

We propose that the interaction between biological sex-at-birth and socially-constructed ‘traditionally genderized traits’ will predict the variance in pain ratings following recent MSK trauma more than either variable in isolation. As such, the aims of this study were to explore the extent that sex-at-birth and three traditionally genderized traits of Relationship-oriented, Goal-oriented, and Emotive explain variance in pain ratings after acute MSK trauma, both in isolation and as an interaction effect. As for the clinical use of these kind of studies, it could be that clinicians and researchers are encouraged to consider both sex- and gender-based variables and have a comprehensive exploration of these differences in their clinical and experimental research.

## Materials and methods

### Participants

Data were drawn from a prospective observational study of recovery following acute non-catastrophic MSK injury (the ‘Systematic Merging of Biology, Mental Health and Environment’ (SYMBIOME) study, clinicaltrials.gov ID number NCT02711085). Methods of screening and recruitment have been described previously [39]. In short, eligible participants were at least 18 years or older, presented to a local urgent care centre for reasons of pain or functional interference following MSK trauma within the prior 3 weeks, did not require inpatient admission or surgery, could read and understand conversational French or English, and did not have a systemic or chronic condition that would be expected to delay recovery such as cancer, neuromuscular disorders or other significant systemic diseases.

At inception (<3 weeks from injury), all participants completed a set of questionnaires that included the Brief Pain Inventory (BPI), the GPES, and a study-specific demographics form including age and sex-at-birth. Participants were followed for up to 12 months post-injury to establish recovery outcomes, though only the baseline data have been used for this analysis. The protocol was approved by the local institutional review board prior to initiating data collection, and participants provided written informed consent prior to participation.

### Study tools

The BPI is an 11-item self-administered questionnaire with subscales to assess Pain Severity and Pain Interference with daily activity [40]. Each of the 11 items is scored on a 10 point numeric rating scale where 0 = no interference/pain and 10 = severe or complete interference/pain. A combination of 4 items form a *pain severity* subscale assess pain at its “worst,” “least,” “average,” and “current pain” (mean /10), and 7 items form a *pain interference* subscale assesses the extent to which pain interferes with general activity, normal work, walking ability, mood, sleep, relationships, and enjoyment of life (sum /70). Higher scores indicate worse pain and more significant functional interference, respectively. The BPI is one of the most widely used pain-related self-reported outcomes tools and has shown acceptable psychometric properties in a variety of conditions, including MSK disorders [41, 42].

The GPES was recently developed by our group as a new gender-focused pain-related expectations and beliefs scale. The first section of the GPES was used in this study; this section includes 16 items that were partly adapted from the Bem Sex Role Inventory (BSRI) [43] considered as traditionally genderized personality characteristics [44]. Through rigorous conceptual and statistical analysis, a three-factor structure was identified. Structural analysis of this 16-item section was conducted through ML(maximum likelihood)-based Confirmatory Factor Analysis (CFA). The factors were termed “Emotive,” “Relationship-Oriented,” and “Goal-Oriented.” [38] Evidence of construct validity was supported through significant sex-based differences (p≤0.02) in the expected directions for all 3 subscales. The self-perceived traits of the GPES seem to allow gender to be explored in new ways in pain research. Participants rate themselves on each item according to how well that trait describes them (“1 = not at all like me,” “2 = somewhat like me,” and “3 = extremely like me”). In a prior analysis of 198 responses to this section, three factors emerged: *Relationship-oriented* (nurturing, giving, and gentle, scored out of 9), *Emotive* (sensitive and emotional scored out of 6), and *Goal-oriented* (determined, leader, competitive, tough, and confident scored out of 15) [38]. The GPES also includes a second independent section in which respondents rate their sensitivity to, endurance for, and willingness to report pain in comparison to others of their same sex, but only the genderized traits section of the tool has been used here.

## Analysis

### Pre-analysis: Descriptives and bivariate associations

Participants’ characteristics were evaluated descriptively (mean ± SD and range/proportion as appropriate). The assumptions of data normality were then examined prior to the association analysis, and the homogeneity of variances was assessed via Levene’s test [45]. As the database represented a mix of extremities (arms or legs) and spinal (neck, upper, or low back) injuries, we first classed all participants according to the primary region of injury (extremity or spinal) and conducted independent t-tests for each of the primary variables to identify any systematic bias in scores. If a significant difference was found, the type of injury was retained in subsequent analyses. In the base analyses, the dependent variables (mean BPI Pain Severity and Interference scores) were evaluated for differences between sex-at-birth (male vs. female) through independent samples t-test or Mann-Whitney U-test, dependent on data distribution. The associations between each of the three GPES subscales and the two dependent variables (BPI Pain Severity and Interference) were evaluated using Pearson’s r. This analysis was also repeated for the sample when disaggregated by sex. As an exploratory analysis intended to inform and help interpret the subsequent multivariate analyses, no a priori hypotheses were posed and no adjustment of p-values was done.

### Hierarchical multivariate linear regression

Stepwise hierarchical multivariate linear regression was used to explore the role of each of the three GPES subscales and sex-at-birth in predicting significant unique variance in BPI Pain Severity and BPI Pain Interference scores. Assumptions of regression (normality, non-multicollinearity, and homoscedasticity) were first evaluated. Normality was tested through the evaluation of histograms, skewness and kurtosis statistics. Nonmulticollinearity was assessed through a correlation matrix (flagging any inter-item correlations >0.85) and the variance inflation factor (VIF) (flagging VIF >3.3) [46–48]. Homoscedasticity was evaluated through inspection of a plot of predicted to residual estimates to ensure no systematic bias or pattern of residuals existed [49, 50].

Three models were created for each of the BPI Pain Severity and BPI Pain Interference scores by forcing all variables into the equation, meaning six total models: Model 1 = Sex, Relationship-oriented score, Model 2 = Sex, Goal-oriented score, and Model 3 = Sex, Emotive score. The final model fit was explored through R2, adjusted R2 and P-value analysis. Finally, the hypothesis of sex ✖ GPES interaction effects was tested for each of the six models established in the previous step by adding the interaction term after including the two independent variables separately. The final model fit was explored through R2, adjusted R2 and P-value analysis.

### Sample size

Sample size was estimated using the algorithms of Soper [51], with 3 predictors and medium effect size (f2) of 0.15, while accepting 5% alpha error and 20% beta error (p<0.05 and 80% power, respectively) the minimum sample was estimated at a minimum of 76 participants.

## Results

### Participant characteristics

A total of 130 participants were recruited in this cohort, of which 113 provided complete data for all of the studied variables. The characteristics of this sample are presented in **Table 1**. The sample was 60.2% female, with a mean age of 43.2 years. Average pain severity and interference ratings were 4.3/10 (SD = 2.0) and 26.9/70 (SD = 16.7), respectively.

**Table 1:** Characteristics of the study sample.

N = 113	
Sex (% female)	60.2%
Age (y), $\bar{x}$ ± SD (min-max) (N = 93)	43.2 ± 14.8 (18–66)
Medicolegal involvement	
None	89.4%
Active insurance claim^1^	10.6%
Region affected	
Axial (neck or back)	26.5%
Peripheral (upper or lower extremities)	73.5%
BPI^2^ subscales, $\bar{x}$ ± SD (min-max), skewness and kurtosis	
Severity (/10)	4.3 ± 2.0 (0–8), -0.07 and -0.89
Interference (/70)	26.9 ± 16.7 (0–67), 0.43 and -0.73

^1^ Includes auto insurance, worker’s compensation, or personal injury claim

^2^ BPI (short form): Brief Pain Inventory

### Pre-analysis

No significant differences in pain severity or interference were found between the two types of injury (spinal vs. extremity, p > 0.07). Data were adequately normal with equal variances in pain between the sex groups. There were 45 males and 68 females, and an independent sample t-test revealed no differences in BPI Pain Severity or Interference between the sexes (**Table 2**).

**Table 2:** Independent sample t-test.

	Male		Female		P-value	95% Confidence Interval of the Difference (LLCI, ULCI)
	N	Mean (SD)	N	Mean (SD)		
**BPI Pain Severity** ^**1**^	45	4.0 (2.1)	68	4.5 (2.0)	0.2	-1.3, 0.2
**BPI Pain Interference** ^**1**^	45	26.4 (16.0)	68	26.8 (17.3)	0.8	-6.8, 5.9

^1^: BPI (short form): Brief Pain Inventory

### Bivariate correlations

**Fig 1:**
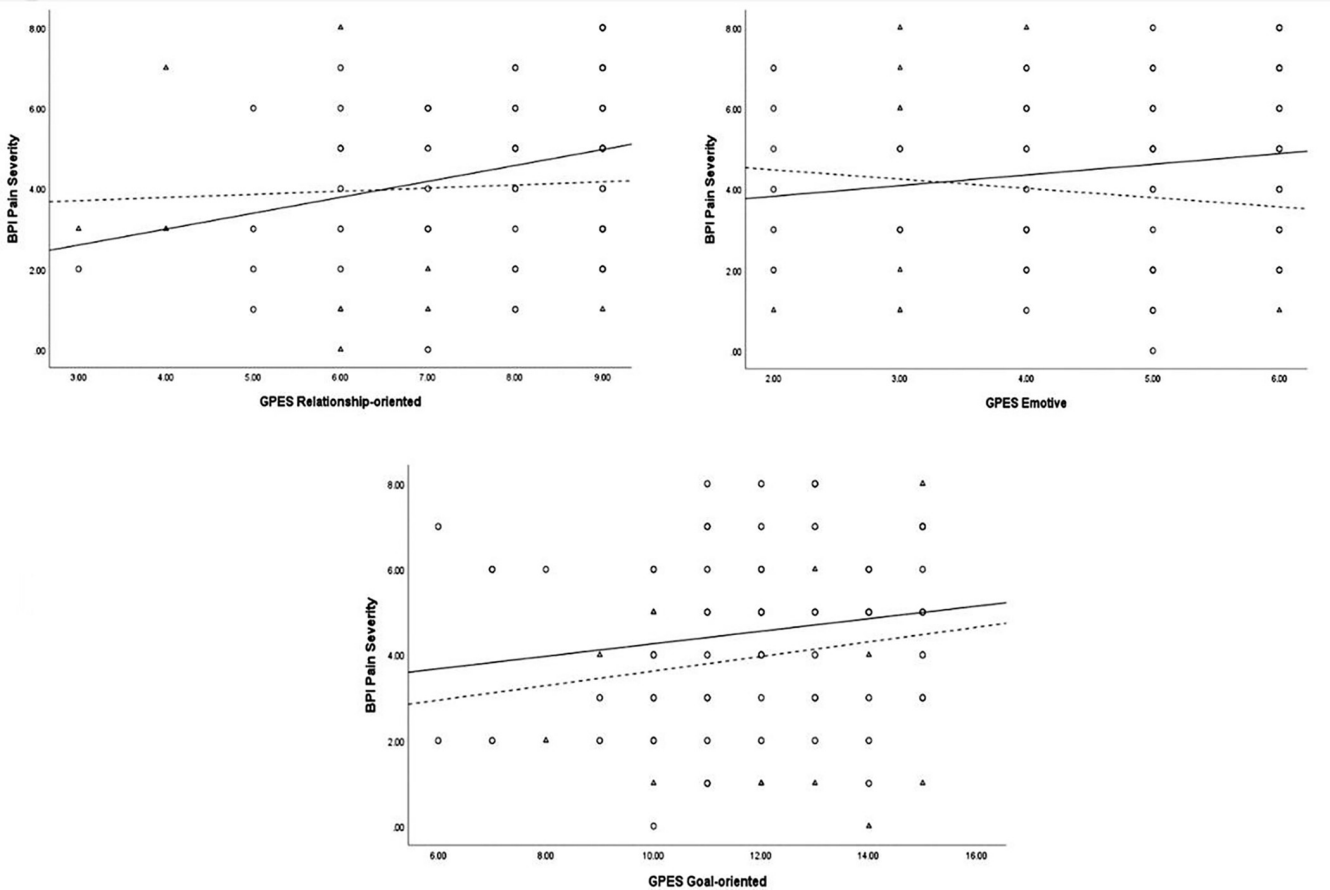
Scatter plot of the relationship between BPI Pain Severity and Relationship-oriented, Emotive, and Goal-oriented subscales in males and females. (○) and (⚊) represent Females; (Δ) and (⚋) represent Males.

**Fig 2:**
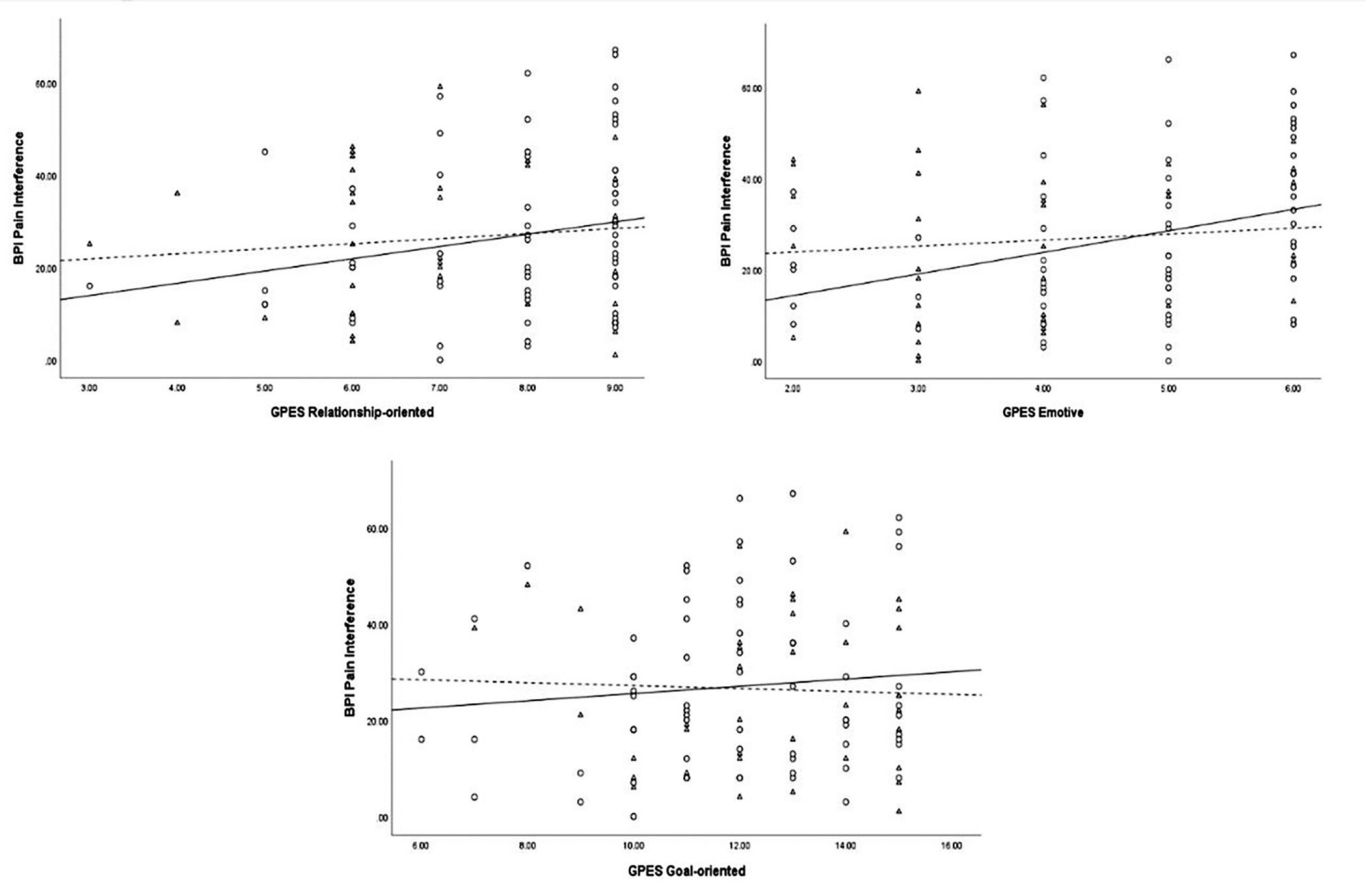
Scatter plot of the relationship between BPI Pain Interference and Relationship-oriented, Emotive, and Goal-oriented subscales in males and females. (○) and (⚊) represent Females; (Δ) and (⚋) represent Males.

**Table 3** contains correlations between GPES subscales and the BPI subscales, both as an overall sample and when stratified by sex. Significant associations were found between the GPES subscale ‘Relationship-oriented’ and BPI Pain Severity (r = 0.20, p < 0.05), and between GPES ‘Emotive’ subscale and BPI Pain Interference (r = 0.24, p < 0.01). In the sex-disaggregated analysis, both the GPES Relationship-oriented and GPES Emotive subscales showed a significant association, respectively, with BPI Pain Severity (r = 0.27, p < 0.05) and BPI Pain Interference (r = 0.33, p < 0.01) scores only in female participants. The GPES Goal-oriented subscale was not associated with either BPI subscales, either as an aggregate sample or when disaggregated by sex. **Figs 1** and **2** provides a graphical representation of the associations between each of BPI Pain Severity and BPI Pain Interference with the independent variables for both males and females, respectively.

**Table 3:** Simple bivariate associations between GPES subscales and pain variables in the acute stage of injury.

	Relationship-oriented	Goal-oriented	Emotive
BPI Pain Severity subscale^1^			
Full sample	**0.20** *	0.15	0.06
Male (n = 45)	0.05	0.16	-0.13
Female (n = 68)	**0.27** *	0.16	0.15
BPI Pain Interference subscale^1^			
Full sample	0.16	0.05	**0.24** **
Male (n = 45)	0.10	-0.04	0.10
Female (n = 68)	0.21	0.10	**0.33** **

*: Correlation is significant at the p < 0.05 level

**: Correlation is significant at the p < 0.01 level

^1^ BPI (short form): Brief Pain Inventory

### Hierarchical multivariate linear regression

Assumptions of regression (normality, linearity, and outliers) were satisfied. No standardized residuals were beyond +/- 3.0 SD from the mean and VIF values were all < 3.3. **Tables 4** and **5** provide the regression results. After partialling out the effects of sex-at-birth, only the GPES Emotive subscale significantly (P = 0.01) predicted BPI Pain Interference, explaining a unique 5.9% of score variance. There were no significant sex ✖ GPES interactions found in any model tested.

**Table 4:** Summary of regression analysis for predicting pain severity by sex and GPES subscales variables.

	β(ϐ)	Δr^2^	ΔF (p)
**Model 1: Relationship-oriented**			
Constant	3.51**		
Sex	0.50 (0.12)	0.01	1.62 (0.20)
Constant	1.88		
Relationship-oriented	0.25 (0.18)	0.03	3.58 (0.06)
Constant	5.52		
Sex x Relationship-oriented	0.31 (0.74)	0.01	1.38 (0.24)
**Model 2: Goal-orientated**			
Constant	3.51**		
Sex	0.50 (0.12)	0.01	1.62 (0.20)
Constant	1.52		
Goal-oriented	0.15 (0.16)	0.02	3.13 (0.07)
Constant	1.04		
Sex x Goal-oriented	-0.02 (-0.07)	0.00	0.01 (0.89)
**Model 3: Emotive**			
Constant	3.51**		
Sex	0.50 (0.12)	0.01	1.62 (0.20)
Constant	3.31**		
Emotive	0.06 (0.03)	0.00	0.14 (0.70)
Constant	6.57**		
Sex x Emotive	0.49 (0.79)	0.02	2.37 (0.12)

*: Correlation is significant at the p < 0.05 level

**: Correlation is significant at the p < 0.01 level

**Table 5:** Summary of regression analysis for predicting pain interference by sex and GPES subscales variables.

	β(ϐ)	Δr^2^	ΔF (p)
**Model 1: Relationship-oriented**			
Constant	25.96**		
Sex	0.46 (0.01)	0.00	0.02 (0.88)
Constant	13.34		
Relationship-oriented	1.95 (0.17)	0.02	3.17 (0.07)
Constant	31.13		
Sex x Relationship-oriented	1.55 (0.44)	0.00	0.49 (0.48)
**Model 2: Goal-orientated**			
Constant	25.96**		
Sex	0.46 (0.01)	0.00	0.02 (0.88)
Constant	21.01*		
Goal-oriented	0.38 (0.05)	0.00	0.28 (0.59)
Constant	42.37		
Sex x Goal-oriented	1.05 (0.42)	0.00	0.48 (0.48)
**Model 3: Emotive**			
Constant	25.96**		
Sex	0.46 (0.01)	0.00	0.02 (0.88)
Constant	15.09*		
Emotive	3.30 (0.25)*	0.06	**6.89 (0.01)**
Constant	37.49*		
Sex x Emotive	3.40 (0.67)	0.01	1.79 (0.18)

*: Correlation is significant at the p < 0.05 level

**: Correlation is significant at the p < 0.01 level

## Discussion

The purpose of this study was to determine whether sex-at-birth and traditionally genderized traits as measured through self-ratings on the GPES subscales could significantly explain variance in scores on the popular BPI questionnaire in people with acute MSK injuries. Findings from this study are in accordance with current conceptualizations of the complexity of pain, and appear to support the inclusion of personal characteristics that may influence pain reporting.

Prior studies have shown some consistencies with part of our results regarding no significant sex differences in either pain severity or pain interference [52–54]. Zhao and colleagues found no differences in BPI pain severity or interference between sexes in a sample of 377 participants with cancer-related pain [52]. Similarly, Chung and colleagues used a case-control design and found no significant sex differences in BPI Pain Severity, Interference and total scores from a survey of 2021 construction workers in different trades [53]. Also a systematic review by Racine et al., 2012, showed that most of the included studies that measured pain unpleasantness and intensity found no sex differences in many pain modalities [10].

However, other studies have found higher scores in the BPI Pain Severity and BPI Pain Interference in women compared to men [1, 55]. Several factors may account for this inconsistency, the most important ones could be the different populations studied, causes of pain, their dominant culture, and the measurement tools used. For instance, in comparison to our study of people with acute MSK injuries, in the study by Stubbs et al. (2010), primary care patients were enrolled who had chronic MSK pain (low back, hip, and knee pain) while half of them had clinical depression as well [1]. Ours is also a rare study in this field focussed on a clinical sample with acute pain, while much of the work in this field has used either clinical samples with chronic pain or healthy samples and experimental pain protocols. It is possible that differences between the sexes in clinical samples manifest more clearly as pain persists.

Robinson et al. highlighted the critical role of gender-specific expectations in explaining sex differences in pain outcomes [56]. In their study, prior to performing the cold pressor task, pain-related gender-role expectations were manipulated by assigning participants to three groups with different instructional sets including no expectation, 30-second gender expectation (“The typical *man/woman* lasts 30 seconds in this task.”), and 90-second gender expectation (“The typical *man/woman* lasts one minute and thirty seconds in this task.”). They showed that sex differences in pain threshold, tolerance and ratings could be altered by manipulating the gender-specific tolerance expectations [56].

The role of psychological and social factors in pain experiences has received considerable attention among pain researchers [19, 57, 58]. There is convincing evidence that both psychological and social factors such as cognitions and emotions, personality traits [22, 59], coping styles [60–62], social, and gender-related norms should be taken into account in pain research. These factors have functioned as both predictors and mediators of pain-related variables [63–66]. In this field, gender has often been conflated with sex, though it is a complex multidimensional construct. Theories from behavioral psychology, such as Social Learning Theory [67] and Cognitive-Developmental Theory [68] suggest that pain perception and expression could be at least partly influenced by gender socialization [69]. For instance, considering social learning theory, some pain models shape at an early age by observing others’ responses to pain, and this could have an important role in understanding the complex pain behavior patterns in adults [70].

According to our results, participants who rated themselves higher on the GPES Relationship-oriented subscale (the combination of nurturing, giving, and gentle items) also reported greater pain severity. Participants who considered themselves more Emotive (the combination of sensitive and emotional items) reported greater pain-related interference. In sex-disaggregated analyses, the effect was only present in female participants. While mechanisms to explain these findings are purely speculative, it makes intuitive sense that those who see themselves as more emotive and relationship-oriented are also more likely to disclose their experiences of distress, and some research has also suggested that to be more emotionally vulnerable could also be associated with reporting higher pain levels and lower pain tolerance [22, 71]. We found that emotive men were not as likely to report pain as emotive women in our study, a possible linkage could be that women are more likely to experience interpersonal adverse life events, that could manifest as greater emotional vulnerability. Another possible explanation could be that men also experience vulnerability, pain and distress but sometimes in combined with an unwillingness to talk about it or report it. For example, in a study by Thorn et al., (2004), the effects of personality and pain catastrophizing on pain tolerance and pain ratings were examined in 219 students by using the PAQ [22]. According to their results, the tendency to be described as emotionally vulnerable partially accounted for the sex-related differences recorded in pain responsivity and pain catastrophizing (Women’s scores were higher on the both pain catastrophizing scale and cold pressor task VAS ratings) [22].

In the present study, we found no significant relationship between GPES Goal-oriented score (determined, leader, competitive, tough, and confident) and either of the BPI Pain Severity or Interference. To the best of our knowledge, there is no other research that has assessed the relationship between the personality characteristics of the GPES Goal-oriented and pain reports. It is possible that this is simply the presence of a social desirability bias (that people are more likely to rate themselves as goal-oriented), but if that was the case, we’d expect such a bias to also mean lower pain severity ratings (people are less likely to complain of pain to others), and therefore a negative correlation should be present. We would also expect that if the phenomenon was social desirability, the results of the Relationship-oriented and Emotive analysis would also have yielded similar non-significant results. Given the current data, all we can say with confidence is that scores on the 5-item Goal-oriented subscale were not associated with pain severity or interference after MSK trauma in our sample. Further investigation is encouraged.

Counter to our a priori assumption that GPES subscales and sex variables would explain differences in BPI Pain Severity and Interference ratings, in the regression analyses, only the Emotive subscale was retained in predicting the pain interference. This was supported by our results of the correlations, as it showed the Emotive trait was positively associated with BPI Pain Interference following an acute MSK trauma. Although the results of the regression analyses were non-significant for other models, the results were suggestive of the potential prognostic factor of Relationship-oriented for pain severity (F Change = 3.58, p = 0.06), and considering the significant correlation between the Relationship-oriented scores and pain severity, it could be implying that this needs more exploration in the future and may be a function of low statistical power. Also, the differential effects of emotiveness between the sexes on pain reports from the disaggregated analyses could indicate a potential interaction effect despite the non-significant findings of regression analysis. Furthermore, as indicated by our results and given the complexity of psychosocial variables and pain experiences, we should not expect that only sex assigned at birth or GPES subscales would completely explain the variances in pain responsivity.

We acknowledge critiques of gender measurement scales such as the BSRI [72–75], and this is why we have used the GPES scale because it does not rely on gendered norms. However, prior studies have used such scales with their traditional underlying assumptions and explored gender roles in pain. In a study examining the mediator roles of pain appraisal and gender role, assessed by the Extended Personal Attributes Questionnaire (EPAQ), 145 participants completed a cold pressor task [66]. Sanford et al. found only the femininity scale had a significant relationship with sex and pain tolerance time, but it was not a significant moderator of the relation between sex and pain tolerance. These authors also found that the interaction effect was not significant through simultaneously entering sex, femininity, and sex by feminine interaction as predictors in a similar approach to what we have used here. Their results suggested that psychosocial variables, including a combination of positive feminine gender roles (such as awareness of feelings and warmth) and threat appraisals of pain, play an important but complex role in partially mediating the sex and pain relationship [66]. Their results regarding masculinity are similar to what we found in the current study, in which the GPES Goal-oriented subscale (traditionally assumed as a masculine trait) was not significantly related to pain-related outcomes and did not function as a significant moderator contrary to the GPES Emotive. This might be due to the more pronounced relationship between the items of GPES Emotive subscale including sensitive and emotional with a higher tendency to report pain.

Another study assessed the effects of gender-role socialization indicated by BSRI, and systolic blood pressure (SBP) of 104 participants in explaining the relationship between sex and pain report from a cold pressor task [17]. Using a similar hierarchical regression, gender-role socialization was only a significant predictor of pain tolerance, while neither SBP nor gender explained sex differences in pain reports [17]. Differences in methodologies between our study and the aforementioned studies [17, 66] may explain our different results. For instance, BSRI [17], EPAQ, [66] or Gender Role Expectations of Pain (GREP) [76, 77] scales were used to a set of regression analyses in the prior studies to explore the relationship of the sex, gender and pain outcomes, however, we used the GPES subscales. Besides using different pain and gender measure tools, we used a sample drawn from a clinical post-trauma population, whereas most prior studies have used experimental pain stimuli (e.g. cold pressor task) in otherwise healthy participants. In a recent study on exploring pain beliefs within and between the sexes, the importance of considering all clinical pain evaluations for clinical treatment strategies is highlighted as one assessment technique is not completely represent the pain experience [78].

Some limitations to the present study will be addressed here. This study investigated a unique, but narrow, aspect of personal characteristics for gender differences. There is a large array of gender-related traits or personality characteristics that were not investigated. The concepts of goal-oriented, emotive or relationship-oriented can be difficult to define and may be gendered in a way that these could manifest differently for men and women. Gendered expectations of these characteristics can also evolve in societies over time. We also argue that if we are admitting that such characteristics which are indicated as masculine or feminine could exist simultaneously in the same person, then maybe it means that the masculine and feminine dichotomy cannot really hold true. Therefore, classifying people based on these scales may need to be revised, since they could express themselves according to their situation and mood in that specific moment. All of these issues complicate explanatory modelling and we see results such as those presented here as steps towards a broader conceptualization of sex, gender, culture, and other socially-constructed phenomena in pain research. A larger sample size should be recruited for future studies. Also, other potential predictors should include in future research in order to have a clearer picture of the pain outcomes, such as depression, stress, and sleep hygine.

In conclusion, results from the current study highlight that variances in pain outcomes such as pain severity and interference might be explained by considering some of the gender-related personality characteristics as it was shown for self-perceptions of ‘Emotive’ qualities. Therefore, not only researchers but also clinicians are encouraged to have a consideration for sex and gender factors in in their comprehensive evaluations. Also, an implication of this work is that it advocates for considering both sex- and gender-based variables when interpreting patient pain reports. From a broader perspective, biological parameters such as hormones should be considered along with the other important dimensions such as socioeconomic status, age, culture, race, and family’s role for a better understanding of people’s differences in pain responding. We expect these studies, and their results will be encouraging further investigating in the sex-and gender-based pain research.
